# N Balance Studies Emphasize the Superior Protein Quality of Pig Diets at High Inclusion Level of Algae Meal (*Spirulina platensis*) or Insect Meal (*Hermetia illucens*) when Adequate Amino Acid Supplementation Is Ensured

**DOI:** 10.3390/ani8100172

**Published:** 2018-10-03

**Authors:** Carmen Neumann, Susanne Velten, Frank Liebert

**Affiliations:** Department of Animal Sciences, Division Animal Nutrition Physiology, Georg-August-University of Goettingen, Kellnerweg 6, 37077 Goettingen, Germany; carmen.neumann@agr.uni-goettingen.de (C.N.); susanne.velten@agr.uni-goettingen.de (S.V.)

**Keywords:** piglets, growing pigs, N balance, N utilization model, amino acids, protein quality, apparent N digestibility, amino acid efficiency

## Abstract

**Simple Summary:**

This study aimed to evaluate diets with complete substitution of soybean meal (SBM) by an algae meal from *Spirulina platensis* (SM) or partly defatted larvae meal from *Hermetia illucens* (HM) as feed for piglets and growing pigs. Main feed ingredients of the experimental diets were wheat, barley, and SM or HM. The final diets contained 21% (piglets) and 13% (growing pigs) of SM or HM and nitrogen (N) balance studies were applied to measure parameters of apparent N digestibility, complex dietary protein quality, and individual amino acid (AA) efficiency according to the ‘Goettingen approach’. Diets were well accepted by the animals and an extended level of AA supplementation yielded improved dietary protein quality with both of the alternative feed proteins. However, HM based diets provided superior apparent N digestibility.

**Abstract:**

Two age-dependent nitrogen (N) balance studies (average body mass 25 and 60 kg) utilized 16 male castrated piglets and 16 barrows to measure N utilization parameters of diets with complete substitution of SBM by alternative protein sources (SM, HM), but different AA fortifications. Lysine supplementation up to 80% of the recommended lysine (Lys) supply in diets HM (A) and SM (A) yielded similar protein quality data (63.6 ± 2.1 and 63.7 ± 3.4). Surprisingly, only in piglet diet HM (AA) did the extended AA supplementation (Lys, methionine (Met), threonine (Thr)) enhance protein quality (72.8 ± 6.7) significantly (*p* = 0.004). Similar trends were observed in growing pigs. However, when the level of histidine (His) in diet SM (AA) was increased, feed protein quality (71.8 ± 1.3) was significantly (*p* < 0.001) improved indicating the importance of adequate His supply in diets with a complete substitution of SBM by the algae meal (SM) under study. AA efficiency data extend the possibilities to explain the observed responses on protein quality. When an adequate AA balancing in the diet is guaranteed, from nutritional point of view both of the alternative proteins may replace SBM in pig diets.

## 1. Introduction

The world population has been steadily growing in recent decades, and by 2050 a figure of 9.7 billion people is forecasted, coinciding with a 70% increase in the demand for food [1]. With changing consumption patterns, meat shares an increasing part of this growing demand for food [2]. World meat production is expected to increase from 320 million tonnes in 2016 to 360 million tonnes in 2025, with pork meat accounting for 36% of world meat production [3] with a growth rate of 12%. Accordingly, large quantities of feed protein will be needed, and as it currently stands soybean products are the main protein ingredients in pig diets. Therefore, they act as a reference in the hunt for alternative sources to meet future protein demands. However, suitable feed alternatives are still needed [4,5]. Algae production could also be an acceptable alternative, because this type of aquatic biomass grows adequately with only marginal land use [4]. Additionally, insects could be an alternative protein source because they can be reared on grain and the larvae grow rapidly. The microalga *Spirulina platensis* is a prokaryotic multicellular cyanobacterium. Spirulina alga belongs to the photosynthetic organisms and grows only in warm climates with high light intensity. Natural environmental conditions are alkaline salt lakes as well as basic fresh water bodies. The black soldier fly *Hermetia illucens* is a widespread fly and belongs to the family of Stratiomyidae, which is a member of the order Diptera. The larvae of Hermetia are able to utilize a wide range of nutrient sources and develop rapidly between 20 °C and 30 °C. More details about the alternative proteins are presented earlier [6,7,8,9]. Generally, both of the alternative proteins have the potential for being included as a protein source in animal feeds. Currently, there are only a few studies about Spirulina in piglet and pig diets, mostly dealing with health-promoting aspects of the microalga [10,11]. Earlier studies [12,13] examined the growth response of piglets with higher inclusion rates of Spirulina meal (8% to 12%) without negative effects. Hermetia also appears to be an adequate protein source; Newton et al. [14] demonstrated the acceptance of diets containing up to 33% full-fat meal from Hermetia larvae as the main protein source in pig diets. Dankwa et al. [15] substituted 100% of the fishmeal content in weaned piglet diets by non-defatted housefly larvae meal without a reduction in growth. Experiments by Ji et al. [16] on early weaned piglets showed that 5% of plasma proteins in diets can be replaced by various insect meals (mealworm (*Tenebrio molitor*), house fly larvae (*Musca domestica*), and large mealworm (*Zophobas morio*)) and induce no significant deficiencies in feed intake and growth performance. As a part of the multidisciplinary project “Sustainability Transitions in the Food Chain” (supported by the Lower Saxony Ministry of Science and Culture) the current study aimed to measure the protein quality of piglet and fattening pig diets based on N balance studies with a high inclusion of partly defatted Hermetia meal (HM) or Spirulina meal (SM) with a graded extent of AA supplementation.

## 2. Materials and Methods

The N balance studies were conducted at facilities of the Division Animal Nutrition Physiology of Georg-August-University of Goettingen and permitted (03.2016/AZ15/2027) by the Ethics Committee of the Lower Saxony Federal Office for Consumer Protection and Food Safety (LAVES), Germany.

### 2.1. Alternative Protein Sources

The analyzed nutrient composition of the alternative protein sources are summarized in Table 1. The microalgae powder of Spirulina platensis was a sun-dried commercial product obtained from Myanmar and declared to be free of genetically modified organism (GMO), irradiation, pesticides, colorants, preservatives, and additives. As demonstrated by the nutrient composition (Table 1), the lipid fraction was not extracted from the algae meal. The microcystine content was analyzed by an external laboratory (TeLA GmbH, Geestland, Germany) and remained under the detection limit. The insect meal was obtained from a commercial producer (Hermetia Futtermittel GbR, Baruth/Mark, Germany). Black soldier fly larvae were separated from the substrate (rye flour, wheat bran) after 20 days, dried for 14 h at 65 °C to 70 °C and following partial defattening with a screw press were finally ground into a meal. The process of partial defattening is needed to improve the handling of the insect meal as a feed ingredient in compound feeds.

### 2.2. Stock and Husbandry

16 piglets and 16 growing pigs of the same genotype (PIC 408 (Large White × German Landrace)), both male castrated, were raised under farming conditions until 25 kg (origin: Gebrueder Weisskittel KG, Hardegsen Troegen, Germany) and utilized for age dependent N balance studies (average body weight 25 and 60 kg). During the experiments, both piglets and growing pigs were housed in metabolism cages for five days of adaptation and two consecutive collection periods (five days each). Metabolism cages allow the separate quantitative collection of feces and urine from individual pigs. Between N balance periods, pigs were kept in floor pens (3 m^2^) with a controlled supply of standardized mixed feeds (single feeding) according to body weight (BW) development.

### 2.3. Diets and Feeding

Experimental diets were manufactured at the facilities of the Division Animal Nutrition Physiology of the University of Goettingen. In both of the age periods under study (piglets 25 kg average BW; growing pigs 60 kg average BW), the 16 pigs were randomly allotted to four pelleted diets (Table 2). The main feed ingredients were wheat, barley, and HM or SM. Consequently, the final diets contained 21% (piglets) and 13% (growing pigs) SM or HM in order to achieve the recommended dietary crude protein (CP) level (19% piglets, 16% growing pigs). In both of the age periods, diets HM (A) and SM (A) were only supplemented with Lys in order to achieve 80% of the recommended Lys supply [17]. Piglet diets HM (AA) and SM (AA) were fortified with an extended AA supplementation (Lys, Met, Thr) according to the recommended ideal AA ratio [17]. For growing pigs, diet HM (AA) was also supplemented according to the ideal AA ratio; however, diet SM (AA) was treated separately to yield more information about the limiting position of histidine (His) in diets with high inclusion level of algae meal. In the first collection period (diet SM^1^(AA)), only an elevated level of Lys was added, but in the second collection period (diet SM^2^(AA)) His was also supplemented to meet the His recommendation according to National Research Council (NRC) [18] and British Society of Animal Science (BSAS) [19]. Through this procedure it was aimed to demonstrate if His is in the limiting position with diet SM^1^(AA).

As demonstrated by the analyzed nutrient composition of the diets (Table 2), crude protein (CP) content and calculated metabolizable energy (ME) concentration in the dry matter (DM) were very similar within the age periods. Crude fiber (CF) contents ranged between 29.1 and 52.7 g/kg DM (piglet diets) or 30.2 and 52.3 g/kg DM (pig diets). CF contents were generally lower in Spirulina diets due to the fact that Spirulina does not contain cellulose in the cell wall. Crude ash (CA) contents were very similar in piglet (50.3 to 53.2 g/kg DM) and pig (42.1 to 45.2 g/kg DM) diets. Due to the remaining fat content in the partly defatted insect meal, crude fat contents were elevated in HM diets. The defattening of insect meals was conducted under commercial conditions by a screw press.

The AA composition of the experimental piglet diets as related to official recommendations [17,18,19] for selected AAs are presented in Table 3. Regarding the diets HM (A) and SM (A) with a basic level of AA supplementation, it is obvious that the supply of several AAs was below the recommendations. In diet HM (A), Lys, methionine and cysteine (Met + Cys), Thr, and leucine (Leu) are deficient. In diet SM (A), the supply of Lys, Met + Cys, Thr, and His is insufficient. In diets of HM (AA) and SM (AA), an improved dietary AA balance was aimed at making use of an extended level of AA supplementation. Consequently, in diet HM (AA) only Leu remained as a potential limiting AA (6% below recommendation). In diet SM (AA) this was the case for His (30% below the recommendation). The aim was not to compensate these AA deficiencies, rather to yield first experimental data about the dietary AA efficiency of these AAs according to applications of the ‘Goettingen approach’.

A similar procedure was applied for the growing pig diets (Table 4). Accordingly, in diet HM (A) the supply of Lys, Met + Cys, Thr, and Leu remained suboptimal; in diet SM (A) Lys and His were below the recommendations. These deficiencies were mostly balanced in diets HM (AA), SM^1^(AA), and SM^2^(AA). A remarkable limitation remained only in diet SM^1^(AA) where His was 18% below the recommendation. This limitation was aimed to be compensated by adding His in diet SM^2^(AA) and the effect on dietary protein quality should be measurable through the response in the N balance.

### 2.4. Collection and Sampling

Pigs were individually weighed at the beginning of the adaption period, as well as at the beginning and the end of the corresponding collection periods. Feed intake was recorded daily within the collection periods, feces were quantitatively collected twice a day during feeding times and immediately frozen (−20 °C). At the end of the collection periods, individual fecal samples were weighed, homogenized, and sampled for further chemical analyses while frozen. To minimize ammonia losses from urine, 50 mL (piglets) or 60 mL (pigs) of sulfuric acid (30%) was added to ensure an acid pH (<2) during urine collection. Aliquot urine samples (10%) were taken daily, mixed carefully, and sampled for nitrogen analysis.

### 2.5. Chemical Analyses

Dietary ingredients, experimental diets, and excreta were carefully homogenized and analyzed according to the German standards [20]. Before analyses, feed ingredients and feed mixtures were ground to 1 mm dimensions. Nitrogen content of feed and feces was measured by the Dumas method (TruMac^®^, Leco Instrument GmbH, Moenchengladbach, Germany) and the fraction of CP was calculated with a factor of 6.25. The urine samples were analyzed by total nitrogen according to Kjeldahl (Vapodest^®^, Gerhardt GmbH and Co.KG, Koenigswinter, Germany). AA composition of the alternative protein sources was detected by ion-exchange chromatography (Biochrom^®^ 30, Biochrom Ltd. Cambridge, England) using acid hydrolysis without and with an oxidation step for quantitative determination of sulfur-containing amino acids. According to earlier reports [6,7,8,9] the presented dietary AA composition was based on previous ingredient AA analyses according to the German standards [20] ether extracts were analyzed following HCl hydrolysis of the feed samples.

### 2.6. Nitrogen Balance Data

The processing of N balance data was done according to the current applications of the ‘Goettingen approach’ [21,22,23,24,25,26] making use of the exponential N utilization model created by Gebhardt [27]. This modelling procedure for N metabolism of growing animals works superior to traditional protein evaluation parameters who are significantly influenced by the level of individual feed intake. This factor of influence may create misleading conclusions about dietary protein quality.

The basic function is an expression of body N retention dependent on N intake taking into account the feed protein quality:

(1) $$\;NR=NR_{max}T\;\left(1-e^{-b\times NI}\right)\;$$

(2) $$\;ND=NR_{max}T\;\left(1-e^{-b\times NI}\right)-NMR\;$$

whereby, NR = daily N retention (ND + NMR) [mg/BW_kg_^0.67^], ND = daily N deposition or N balance (mg/BW_kg_^0.67^), NMR = daily N maintenance requirement (mg/BW_kg_^0.67^), $NR_{max}T$ = theoretical maximum for daily N retention (mg/BW_kg_^0.67^), b = model parameter for the slope of the function between NI and NR, depending on the dietary protein quality, NI = daily N intake (mg/BW_kg_^0.67^), e = basic number of natural logarithm (ln).

The genotype dependent model parameters for daily NMR (piglets: 433 mgN/BW_kg_^0.67^; pigs: 388 mgN/BW_kg_^0.67^) and $NR_{max}T$ (piglets: 4697 mgN/BW_kg_^0.67^; growing pigs: 3104 mgN/BW_kg_^0.67^) were taken from earlier studies [28,29]. According to recent reports making use of the exponential model [6,7,21,22,23,24,26,30,31,32,33,34,35], the dietary protein quality was evaluated by parameter (b) based on the equation:

(3) $$b=\frac{\left[lnNR_{max}T-\ln\left(NR_{max}T-NR\right)\right]}{NI}$$

Equation (3) is the result of the logarithmization and transformation of Equation (1).

Earlier reports [36,37] have already stated that the concentration (c) of the limiting amino acid (LAA) in the feed protein and the resulting dietary protein quality (model parameter b) are linearly related. In consequence, quotient $bc^{-1}$ defines the slope of the linear relationship and is an expression of the efficiency of the LAA in the dietary protein [38,39,40], taking into account digestion, absorption, and post absorptive AA utilization. Data analysis is based on total AA, but considers the observed AA efficiency.

(4) $$bc^{-1}=\frac{b}{c}$$

where $bc^{-1}$ is the slope between c and  b, indicating the efficiency of LAA utilization; b is the model parameter of dietary protein quality, and c is the concentration of the LAA in the feed protein (g/100 g of CP).

The relationship between c and b is only expressive in terms of AA efficiency in case of a validated limiting position of the AA under study.

Additionally, traditional parameters like the productive protein value (PPV) and net protein utilization (NPU) were applied to evaluate the complex dietary protein quality by taking into account the processes involved in digestion and post-absorptive utilization.

(5) $$PPV\;\left(\%\right)=\frac{ND}{NI}\times 100$$

(6) $$\;NPU\;\left(\%\right)=\frac{NR}{NI}\times 100$$

However, the traditional protein quality measures (PPV and NPU) are not independent of the level of actual protein intake (NI) [28,38,41]. Consequently, a standardization of protein intake was conducted according to Thong and Liebert [38], providing NPU data which are independent of NI [28,32,38].

Accordingly, standardized net protein utilization ($NPU_{std}$) was calculated (Equation (7)) for equal daily nitrogen intake (piglets: $NI_{std}$: 3500 mgN/BW_kg_^0.67^, pigs: $NI_{std}$: 3800 mgN/BW_kg_^0.67^) to ensure the comparability of derived NPU data:

(7) $$\;NPU_{std}\;\left(\%\right)=\frac{NR_{max}T\;\left(1-e^{-bNI_{std}}\right)}{NI_{std}}\times 100$$

Accordingly, $NPU_{std}$ is applied for evaluating the dietary effects on protein quality in this study.

The apparent fecal digestibility of N is calculated according to Equation (8):

(8) $$VQ_{N}\left(\%\right)=\left(\frac{I-F}{I}\right)\times 100$$

whereby, $VQ_{N}$ = Apparent digestibility of N (%), I = Intake of N (gDM/d), F = Output of N through feces (gDM/d).

### 2.7. Statistical Analyses

Statistical analyses were conducted with the SPSS software package (IBM SPSS Statistics, Version 24.0, IBM Corp, Armok, NY, USA) and results are presented as means ± standard deviation. Different superscript letters are indicating significant differences between the experimental diets. One-way analysis of variance (ANOVA) tests were performed to compare means of the primary N balance data. To verify the variance homogeneity and identification of significant differences (*p* ≤ 0.05) the Games-Howell and Tukey tests were applied. Outliers were identified according to the procedure of Dixon and Massey [42].

## 3. Results

The results of the piglet N balance trials are summarized in Table 5. Between the four experimental diets no significant differences in body weight (BW) (*p* = 0.736), feed intake (*p* = 0.224) and N intake (*p* = 0.257) were observed. Fecal N excretions were enhanced (*p* = 0.004) in the diets HM (A) (717 ± 60), SM (A) (841 ± 132) and SM (AA) (745 ± 123). Superior urine N excretion was observed both with diets HM (A) (1087 ± 118) and SM (A) (958 ± 121), which did not have an extended AA supplementation. Total N excretion was lowest with diet HM (AA) (1463 ± 146), but was not significantly different from diet SM (AA) (1584 ± 285). Daily N balance data were also the highest with diet HM (AA) (2182 ± 325), but again were only numerically different between diets (*p* = 0.053). According to the observed fecal N excretion, HM diets yielded superior apparent N digestibility. As compared to diet HM (A), the extended AA supplementation in diet HM (AA) responded significantly (*p* = 0.004) in dietary protein quality (NPU_std_). This effect was less pronounced between the SM diets.

The observed bc^-1^ values (Table 5) are a reflection of the individual AA efficiency and need to be compared between the diets, while contextualized with the degree of limiting position as related to the other AAs under study (Table 3 and Table 4). Between piglet diets, the highest Lys efficiency (35 ± 3) was observed in diet SM (A), but this was only significantly different (*p* = 0.032) from diet SM (AA) (30 ± 4) with its Lys supply according to the recommendations. Superior Met efficiency (137 ± 6) was achieved with diet HM (A) having the lowest Met supply, which indicated that Met was the most limiting AA in this diet. A significant difference in Thr efficiency between diets was not observed, but the observed trends require discussion. The extended degree of AA supplementation in diet HM (AA) provided superior Leu efficiency (37 ± 5) indicating the strong limiting position of Leu in this diet. Accordingly, SM diets yielded a significantly (*p* < 0.001) higher His efficiency (118 ± 9 and 125 ± 18) as compared to HM (73 ± 3 and 91 ± 12) diets, supporting the strong His limitation as calculated (Table 3) in both of the SM diets.

Results of the N balance study with growing pigs (Table 6) also indicate that the diets with basic AA supplementation are inferior as compared to the diets with extended AA fortification. In addition, both of the HM diets created less fecal N excretion (*p* < 0.001) and in consequence have significantly higher (*p* < 0.001) N digestibility compared to the SM based diets. The urine N losses were significantly decreased (*p* < 0.001) both with diet SM^1^(AA) (971 ± 111) and SM^2^(AA) (967 ± 50). Resulting N balance data were similar in diets HM (A) (1718 ± 241), SM (A) (1847 ± 135), HM (AA) (2054 ± 272), and SM^1^(AA) (1915 ± 126). However, due to the addition of L-His, diet SM^2^(AA) created a significantly improved (*p* < 0.001) daily N balance (2411 ± 55). Looking more closely at the NPU_std_, an enhanced protein quality was found with diets on the extended degree of AA supplementation, and the superior protein quality (*p* < 0.001) was achieved in diet SM^2^(AA) (71.8 ± 1.3) due to the compensated His deficiency. Excluding the consideration of the preliminary results in diet SM^2^(AA), diet SM^1^(AA) yielded superior His efficiency (*p* < 0.001), indicating the validated limiting position of His in this diet.

Lys efficiency did not significantly differ amongst the other diets, but the observed trend between diet HM (A) (60 ± 11) and HM (AA) (68 ± 19) was unexpected. Regarding the Met efficiency, the highest bc^−1^ (285 ± 78) value for diet HM (AA) could indicate that Met supply was still nearly limiting in the AA balanced diet HM (AA), but both the observed Thr efficiency and Leu efficiency could reflect a co-limiting situation.

## 4. Discussion

In the current studies, no significant effects were observed for average body weight of piglets or growing pigs. In addition, we found no significant influence on feed intake for piglets, but did observe an effect for growing pigs. However, these parameters were only measured within the time schedule of N balance studies and should not be over-interpreted, but were eliminated for protein quality evaluation by the applied modelling procedure (Goettingen approach). A few previous studies have already demonstrated that SM could be a good protein source in pig diets [10,12,13,43]. Grinstead et al. [10] examined graded contents of SM (2–20 g/kg) in diets for weaned piglets. Pigs that received 20 g Spirulina per kg feed had significantly higher feed intake and higher daily gains than the control group. Pig diets with 12% SM [12], 9% SM [13] or at least half of the protein from soybean meal substituted by algae meal [43] created no adverse effects on growth performance. Other studies have also reported that pig diets containing insect meals are well accepted [14,16,44]. Pig diets with 5% insect meals (*Tenebrio molitor*, *Musca domestica* larvae, and *Zophobas morio*) substituting 5% plasma protein powder [44] or 6% mealworm meal instead of SBM [16] yielded no significant effects on growth performance. 

Regarding the apparent N digestibility observed in our studies (piglets and pig), HM diets achieved superior N digestibility as compared to the SM diets. This supports the conclusion that, in particular, the algae meal of *Spirulina platensis* is more poorly digestible than the insect larvae meal. The level of AA supplementation in the experimental diets was not a factor of significant influence on the observed N digestibility. Nonetheless, Février and Sève [12] demonstrated that digestibility of feed mixtures was somewhat inferior when Spirulina meal was incorporated. Other reports from Martinavičius [45] with a low level of added SM (2 g/d) showed numerically higher daily gains as well as enhanced digestibility of fat, organic matter, and protein. The very low supplementation of Spirulina in this diet could explain the effects on protein digestibility. To our present knowledge, further relevant studies assessing the digestibility of micro algae meals in pig diets are not available. Other aspects influencing the digestibility of SM are discussed elsewhere [7,8,9]. As for the inclusion of insect meals, Newton et al. [14] studied the digestibility of piglet diets with *Hermetia illucens* when substituting 100% of the SBM by a dried larvae meal of *Hermetia illucens*. The reported apparent digestibility of dry matter, nitrogen, ether extract, crude fiber, ash, NFE, calcium, and phosphorus for the larvae meal based diet were 77.5, 76.0, 83.5, 53.8, 45.2, 84.7, 38.9, and 23.0, respectively. The corresponding data for the soybean meal-based diet were 85.3, 77.2, 73.0, 49.2, 61.6, 91.3, 39.3, and 51.3. The observed digestibility of dry matter, nitrogen, ash, and NFE was higher in the plant-based diet. Further aspects about the digestibility of HM are discussed elsewhere [7,8,9].

Looking more closely at the standardized NPU (NPU_std,_) as an N intake independent measure of the feed protein quality, during the piglet period a significantly higher protein quality was observed for the HM (AA) diet compared to diets HM (A) and SM (A). In growing pigs, diets SM^2^(AA) and HM (AA) created superior protein quality. However, the achieved dietary AA balance following extended the AA supplementation was an important factor of influence. Our results have clearly demonstrated that diets composed of the alternative proteins under study with an extended AA supplementation level yielded superior parameters of feed protein quality. This observation is also supported by current reports with chickens from Neumann et al. [6,7] and Velten et al. [9]. The actual AA supplementation aimed to meet the current ideal amino acid (IAAR) assumption [17]. In piglets, the extended AA supplementation with diet HM (AA) yielded a significantly higher protein quality, indicating that an enlarged range of AA supplementation (Lys, Met, Thr) is required. In contrast, diet SM (AA) with extended AA supplementation did not significantly achieve improved feed protein quality compared to diet SM (A). However, the dietary His supply remained 30% below the recommendation of NRC [18] and BSAS [19] and this fact meant that His was the limiting AA. In growing pigs, the extended AA supplementation with diet HM (AA) achieved only a numerical increase in feed protein quality. However, the additional supplementation of His in diet SM^2^(AA) yielded a significant improvement in feed protein quality, indicating that His was indeed the limiting AA in diet SM^1^(AA). Accordingly, the elevated level of Lys in diet SM^1^(AA) could not improve the dietary protein quality, but the supplementation of His in diet SM^2^(AA) provided significantly higher feed protein quality. 

A large variation in the recommended optimal amount of His supply can be observed throughout the literature. The GfE [17] recommendations for piglets are substantially higher (+15%) in comparison to NRC [18] and BSAS [19]. Also, for growing pigs, higher in-feed concentrations (+17–26%) were recommended in the German requirement standards [17]. As a consequence, diet SM^2^(AA) containing 3.68 gHis/kg (Ratio Lys:His = 100:35) supplied 12% more His as recommended by NRC [18] and BSAS [19], but taking into account the German recommendation (Ratio Lys:His = 100:47) His could still be considered the limiting AA. As demonstrated both by the very high level of His efficiency (bc^−1^_His_) and insignificant difference between diets SM^1^(AA) and SM^2^(AA), a further limitation of His in the His supplemented diet was plausible. This is the first validated measure of His efficiency under an approved His limitation in the diet according to the ‘Goettingen approach’. However, the His supplementation unexpectedly affected protein quality leading to significantly improved AA efficiency of each of the AAs under study. As a consequence, the Lys efficiency in diet SM^2^(AA) also exceeded the results of the other diets, but cannot be explained from a physiological point of view and we rank these results as preliminary. Therefore, the recommendation of NRC [18] and BSAS [19] for optimal His supply could be set too low. However, the limited number of repetitions with this diet is only indicating in this direction, but should not be taken as a validating conclusion, yet.

Continuing the discussion on the effect that diet has on AA efficiency, it has to be pointed out that effects only within the age periods and for relative AA supply below 100% are of interest. The methodical background is reported in more detail elsewhere [24,25]. Accordingly, only the most important observations will be discussed further. In piglet diets, the Lys efficiency differed only between the SM diets, indicating that the elevated Lys supply with diet SM (AA) impaired the Lys efficiency due to the remaining low level of His in both of these diets. Accordingly, the His efficiency was superior in SM diets. The high level of Met efficiency in diet HM (A) (exceeding the data of the other diets), clearly indicates Met as the limiting AA in diet HM (A). Otherwise, the lowest Leu supply created a significantly improved Leu efficiency with diet HM (AA). The observed Thr efficiency was not significantly different between diets and in agreement with the fact that this AA was not in a clear first limiting position in any of the diets under study. 

In growing pigs, significantly superior data of AA efficiency were generally observed in diet SM^2^(AA), but this result should not be over-interpreted according to the former discussion related to His efficiency. Lys efficiency was only numerically different between the other diets, indicating that a limiting position of Lys was not verified. Accordingly, Met efficiency did not significantly differ between diets, with the exception of diet SM^2^(AA). In addition, diet HM (AA) produced superior efficiency of both Met and Thr, but the effect was not significant. In the case of Leu efficiency, the response with HM (AA) was more distinct but only significantly different to diet SM (A); although the same relative level of Leu was supplied in both of the HM diets. In summary, the high level of His efficiency as found with the His limiting SM diets in growing pigs is of further interest to generate the first AA efficiency data under conditions of a validated limiting position for His using the ‘Goettingen approach’.

Finally, further investigations are required to optimize the dietary AA balance in pig diets with an elevated inclusion level of the alternative feed proteins under study. However, up to now it can be ascertained that both protein sources are useful from the viewpoint of dietary protein quality when an appropriate AA supplementation is applied. Actually, insect-based meals are still not authorized for pig feeds in the European Union [46]. Nonetheless, the EU legislative barriers are expected to be overcome in the near future according to the permission for aquafeed [47]. However, the profitability of the two alternative protein sources needs to be improved. 

The general conclusion of this study is that partly defatted insect meal from *Hermetia illucens* (HM) larvae or the microalgae *Spirulina platensis* (SM) at inclusion rates of 21% (piglets) and 13% (pigs) are useful protein alternatives in diets for piglets and growing pigs. The high inclusion rate of SM and HM was applied to demonstrate their potential; however, limitations were uncovered when the dietary AA balance is not sufficiently supplemented. In this context, we aimed to quantify these responses on dietary protein quality by means of N balance studies.

## 5. Conclusions

High inclusion levels of partly defatted Hermetia meal or Spirulina meal in piglet and pig diets depressed dietary protein quality when amino acid supplementation was incomplete, as demonstrated with Lys at 80% of its recommendation. With an extended level of AA supplementation, the observed protein quality of diets with both alternative proteins was improved. Diets with Hermetia meal yielded significantly higher apparent N digestibility compared to diets with Spirulina meal. Nonetheless, both partly defatted *Hermetia illucens* and alga meal from *Spirulina platensis* are promising alternative protein sources in piglet and pig diets, so long as the dietary AA balance is well-adapted to the IAAR recommendations through an enlarged range of supplemented feed AAs. Further investigations are needed to exploit the complete potential of AA supplementations in diets with a high inclusion level of alternative protein sources so that AA efficiency data may provide additional information to optimize the dietary AA balance.

## Figures and Tables

**Table 1:** Analyzed crude nutrient composition (% of DM) and amino acid content of *Spirulina platensis* and *Hermetia illucens* meals as applied for diet formulation.

Nutrient Content	Spirulina Meal (SM)		Hermetia Meal (HM)	
Moisture (%)	3.4		5.5	
Crude protein	58.8		60.8	
Crude ash	6.1		7.5	
Crude lipids	4.3		14.1	
Crude fiber	0.5 *		10.9	
**AA Contents**	**mgAA/gDM**	**gAA/16gN**	**mgAA/gDM**	**gAA/16gN**
Lys	22.97	3.91	32.97	5.42
Met	10.61	1.81	7.53	1.24
Cys	4.53	0.77	4.89	0.80
Thr	25.77	4.39	21.70	3.57
Arg	39.92	6.79	25.05	4.12
Val	34.50	5.87	32.58	5.36
Leu	47.23	8.04	37.95	6.24
IIe	29.81	5.07	23.47	3.86
His	7.51	1.28	16.58	2.73

DM: dry matter; AA: amino acid; N: nitrogen. * Preliminary data due to difficulties in application of the standard procedure.

**Table 2:** Ingredient composition (g/kg as-fed) and analyzed crude nutrients (g/kgDM).

Ingredients/Diets	Piglets (25 kg)				Growing Pigs (60 kg)				
	HM (A)	SM (A)	HM (AA)	SM (AA)	HM (A)	SM (A)	HM (AA)	SM ^1^(AA)	SM ^2^(AA)
**Ingredients (g/kg as-fed)**									
Wheat	339.2	347.4	336.2	345.2	400.7	405.7	399.1	404.5	404.0
Barley	339.2	347.4	336.2	345.2	400.7	405.7	399.1	404.5	404.0
Spirulina meal	-	210.0	-	210.0	-	130.0	-	130.0	130.0
Hermetia meal	210.0	-	210.0	-	130.0	-	130.0	-	-
Soybean oil	80.0	62.0	80.0	62.0	46.0	35.0	46.0	35.0	35.0
Premix ^1^	15.0	15.0	15.0	15.0	10.0	10.0	10.0	10.0	10.0
CaCO_3_	10.0	10.0	10.0	10.0	8.0	8.0	8.0	8.0	8.0
NaCl	1.0	1.0	1.0	1.0	-	-	-	-	-
Titanium dioxide	3.0	3.0	3.0	3.0	3.0	3.0	3.0	3.0	3.0
L-Lysine∙HCl	2.7	4.3	6.1	7.6	1.7	2.7	4.2	5.1	5.1
DL-Methionine	-	-	1.4	0.7	-	-	0.2	-	-
L-Threonine	-	-	1.1	0.3	-	-	0.4	-	-
L-Histidine	-	-	-	-	-	-	-	-	1.0
**Crude nutrients (g/kgDM)**									
Crude protein	210.7	221.8	227.3	222.1	182.8	189.0	178.7	181.2	186.7
Crude fat	133.7	100.1	139.6	99.1	91.8	69.7	95.0	68.4	65.4
Crude fiber	52.7	29.1	48.1	30.6	45.1	38.9	52.3	39.6	30.2
Crude ash	52.7	51.1	53.2	50.3	44.2	42.1	45.2	43.0	42.4
N-free extract	550.2	597.9	531.8	597.9	636.1	660.3	628.8	667.8	675.3
ME (MJ/kgDM) ^2^	17.1	17.0	17.1	17.0	16.4	16.3	16.4	16.3	16.3

HM (A): Hermetia meal with Lys (80%); SM (A): Spirulina meal with Lys (80%); HM (AA): Hermetia meal with extended AA supply; SM (AA): Spirulina meal with extended AA supply; SM^1^(AA): Spirulina meal with extended AA supply collection period 1; SM^2^(AA): Spirulina meal with extended AA supply collection period 2. ^1^ Supplementation of piglet diets (per kg of final diet): Ca, 0.18%; P, 0.21%; Na, 0.008%; Mg, 0.01%; vitamin A, 9000 IU; vitamin D3, 1050 IU; vitamin E, 52.5 mg; thiamine, 1.5 mg; riboflavin, 4.5 mg; vitamin B6, 3.8 mg; vitamin B12, 30 µg; vitamin K3, 3 mg; nicotinic acid, 18.8 mg; calcium pantothenate, 11.3 mg; folic acid, 1.1 mg; biotin, 225 µg; choline chloride, 525 mg; iron, 150 mg; copper, 30 mg; manganese, 37.5 mg; zinc, 150 mg; iodine, 0.23 mg; selenium, 0.23 mg; phytase (EC 3.1.3.8), 501 FTU; supplementation of diets for growing pigs (per kg of final diet): Ca, 0.14%; P, 0.10%; Na, 0.12%; vitamin A, 4000 IU; vitamin D3, 500 IU; vitamin E, 40 mg; thiamine, 1.5 mg; riboflavin, 6.0 mg; vitamin B6, 3 mg; vitamin B12, 30 µg; vitamin K3, 3 mg; nicotinic acid, 20.0 mg; calcium pantothenate, 12.0 mg; folic acid, 0.5 mg; biotin, 100 µg; choline chloride, 100 mg; iron, 80 mg; copper, 5 mg; manganese, 27.5 mg; zinc, 75 mg; iodine, 0.68 mg; selenium, 0.2 mg; phytase (EC 3.1.3.8), 500 FTU. ^2^ Metabolizable energy (ME), calculated according to fed calculation program Hybrimin^®^ (Vers. 8, Hybrimin^®^ GmbH & Co. KG, Hessisch Oldendorf, Germany) on analyzed crude nutrient content.

**Table 3:** Recommended and achieved AA supply ^1^ (in g/kg as-fed resp. in percent of the recommendation) in piglet diets under study.

	Lys	Met + Cys	Thr	Val	Leu	IIe	His ^3^
Recommendation ^2^	12.70 (100)	6.35 (100)	7.62 (100)	7.87 (100)	12.70 (100)	6.22 (100)	4.32 (100)
HM (A)	10.94 (86)	5.14 (81)	6.52 (86)	9.46 (120)	11.95 (94)	6.88 (111)	4.84 (112)
SM (A)	10.15 (80)	5.70 (90)	7.31 (96)	9.81 (125)	13.77 (108)	8.12 (131)	3.02 (70)
HM (AA)	13.57 (107)	6.49 (102)	7.58 (100)	9.43 (120)	11.91 (94)	6.86 (110)	4.82 (112)
SM (AA)	12.71 (100)	6.37 (100)	7.59 (100)	9.97 (127)	13.74 (108)	8.10 (130)	3.01 (70)

AA: amino acid; HM (A): Hermetia meal with Lys (80%); SM (A): Spirulina meal with Lys (80%); HM (AA): Hermetia meal with extended AA supply; SM (AA): Spirulina meal with extended AA supply. ^1^ Derived from analyzed AA content of the ingredients. ^2^ According to Gesellschaft für Ernährungsphysiologie (GfE) [17]. ^3^ According to NRC [18] and BSAS [19].

**Table 4:** Recommended and achieved AA supply ^1^ (in g/kg as-fed resp. percent of the recommendation) in growing pig diets.

	Lys	Met + Cys	Thr	Val	Leu	IIe	His ^3^
Recommendation ^2^	9.40 (100)	4.79 (100)	5.64 (100)	6.11 (100)	9.87 (100)	4.61 (100)	3.29 (100)
HM (A)	8.03 (85)	4.66 (97)	5.25 (93)	7.49 (123)	9.81 (99)	5.47 (119)	3.83 (116)
SM (A)	7.55 (80)	5.01 (105)	5.73 (102)	7.70 (126)	10.94 (111)	6.24 (135)	2.71 (82)
HM (AA)	9.97 (106)	4.85 (101)	5.63 (100)	7.47 (122)	9.79 (99)	5.46 (118)	3.83 (116)
SM^1^(AA)	9.41 (100)	5.00 (104)	5.73 (102)	7.69 (126)	10.93 (111)	6.23 (135)	2.71 (82)
SM^2^(AA)	9.41 (100)	5.00 (104)	5.72 (102)	7.68 (126)	10.92 (111)	6.23 (135)	3.68 (112)

AA: amino acid; HM (A): Hermetia meal with Lys (80%); SM (A): Spirulina meal with Lys (80%); HM (AA): Hermetia meal with extended AA supply; SM (AA): Spirulina meal with extended AA supply; SM^1^(AA): Spirulina meal with extended AA supply collection period 1; SM^2^(AA): Spirulina meal with extended AA supply collection period 2. ^1^ Derived from analyzed AA content of the ingredients. ^2^ According to GfE [17]. ^3^ According to NRC [18] and BSAS [19].

**Table 5:** Summarized results of the N balance study with piglets (BW 25 kg)

Diets	HM (A)	SM (A)	HM (AA)	SM (AA)	SEM	*p*
*n*	7 ^1^	8	8	8		
Mean BW (kg)	25.7 ± 2.5	25.5 ± 3.1	26.5 ± 2.2	25.1 ± 2.0	0.433	0.736
DM intake (g/d)	944 ± 103	928 ± 87	935 ± 86	832 ± 168	21.536	0.224
N intake (mg/BW_kg_^0.67^/d)	3675 ± 179	3666 ± 274	3645 ± 226	3361 ± 579	65.224	0.257
N excretion feces (mg/BW_kg_^0.67^/d)	717 ^ab^ ± 60	841 ^b^ ± 132	620 ^a^ ± 94	745 ^ab^ ± 123	23.388	0.004
N excretion urine (mg/BW_kg_^0.67^/d)	1087 ^b^ ± 118	958 ^ab^ ± 121	843 ^a^ ± 155	839 ^a^ ± 172	30.553	0.007
N excretion total (mg/BW_kg_^0.67^/d)	1805 ^b^ ± 108	1799 ^b^ ± 157	1463 ^a^ ± 146	1584 ^ab^ ± 289	41.916	0.040
N balance (mg/BW_kg_^0.67^/d)	1870 ± 121	1867 ± 199	2182 ± 325	1777 ± 420	57.647	0.053
Apparent N digestibility (%)	80.5 ^ab^ ± 1.8	77.1 ^a^ ± 2.7	82.9 ^b^ ± 3.2	77.7 ^a^ ± 2.7	0.625	0.001
Model parameter b (×10^6^) ^2^	184 ^a^ ± 8	184 ^a^ ± 14	225 ^b^ ± 30	191 ^ab^ ± 27	4.884	0.003
NPU_std_ (%) ^3^	63.6 ^a^ ± 2.1	63.7 ^a^ ± 3.4	72.8 ^b^ ± 6.7	65.2 ^ab^ ± 6.6	1.124	0.004
AA efficiency						
bc^−1^_Lys_ (×10^6^)	32 ^ab^ ± 1	35 ^b^ ± 3	33 ^ab^ ± 4	30 ^a^ ± 4	0.685	0.032
bc^−1^_Met_ (×10^6^)	137 ^b^ ± 6	112 ^a^ ± 8	112 ^a^ ± 15	97 ^a^ ± 14	3.194	<0.001
bc^−1^_Thr_ (×10^6^)	54 ^b^ ± 3	49 ^ab^ ± 4	58 ^b^ ± 8	50 ^ab^ ± 7	1.206	0.026
bc^−1^_Leu_ (×10^6^)	30 ^b^ ± 1	26 ^a^ ± 2	37 ^c^ ± 5	27 ^ab^ ± 4	0.982	<0.001
bc^−1^_His_ (×10^6^)	73 ^a^ ± 3	118 ^c^ ± 9	91 ^b^ ± 12	125 ^c^ ± 18	4.275	<0.001

BW: body weight; DM: dry matter; NPU_std_: standardized net protein utilization; HM (A): Hermetia meal with Lys (80%); SM (A): Spirulina meal with Lys (80%); HM (AA): Hermetia meal with extended AA supply; SM (AA): Spirulina meal with extended AA supply; SEM: standard error of the mean; *p*: *p*-value; ^a–c^ values within a row with different superscripts are significantly different (*p* < 0.05). ^1^ Outlier (α= 0.05) according to Dixon and Massey [42]. ^2^ Applied for NPU standardization based on: NMR = 433 mg/BW_kg_^0.67^/d; NRmaxT = 4697 mg/BW_kg_^0.67^/d. ^3^ Standardized daily N intake = 3500 mg/BW_kg_^0.67^.

**Table 6:** Summarized results of the N balance study with growing pigs (BW 60 kg).

Diets	HM (A)	SM (A)	HM (AA)	SM^1^(AA)	SM^2^(AA)	SEM	*p*
*n*	8	8	8	4	3 ^1^		
Mean BW (kg)	60.5 ± 4.8	58.4 ± 3.8	61.8 ± 3.2	56.3 ± 3.8	60.6 ± 4.5	0.744	0.206
DM intake (g/d)	2064 ^ab^ ± 186	2106 ^b^ ± 172	2109 ^b^ ± 188	1864 ^a^ ± 23	2229 ^b^ ± 79	32.464	0.073
N intake (mg/BW_kg_^0.67^/d)	3769 ^ab^ ± 252	3957 ^ab^ ± 255	3850 ^ab^ ± 238	3642 ^a^ ± 151	4179 ^b^ ± 56	46.477	0.036
N excretion feces (mg/BW_kg_^0.67^/d)	604 ^a^ ± 79	810 ^b^ ± 49	586 ^a^ ± 69	756 ^b^ ± 96	801 ^b^ ± 51	21.749	<0.001
N excretion urine (mg/BW_kg_^0.67^/d)	1447 ^b^ ± 199	1301 ^b^ ± 144	1210 ^ab^ ± 231	971 ^a^ ± 111	967 ^a^ ± 50	43.150	<0.001
N excretion total (mg/BW_kg_^0.67^/d)	2052 ^ab^ ± 241	2110 ^b^ ± 176	1796 ^ab^ ± 239	1726 ^a^ ± 189	1767 ^ab^ ± 1.5	45.170	0.007
N balance (mg/BW_kg_^0.67^/d)	1718 ^a^ ± 241	1847 ^a^ ± 135	2054 ^ab^ ± 272	1915 ^a^ ± 126	2411 ^b^ ± 55	50.149	<0.001
Apparent N digestibility (%)	84.0 ^b^ ± 1.5	79.1 ^a^ ± 1.2	84.8 ^b^ ± 1.5	79.3 ^a^ ± 1.9	80.8 ^a^ ± 1.0	0.496	<0.001
Model parameter b (×10^6^) ^2^	307 ^a^ ± 55	324 ^a^ ± 27	424 ^a^ ± 116	375 ^a^ ± 44	558 ^b^ ± 36	18.166	<0.001
NPU_std_ (%) ^3^	55.7 ^a^ ± 5.6	57.7 ^a^ ± 2.5	64.1 ^ab^ ± 6.6	61.8 ^a^ ± 3.4	71.8 ^b^ ± 1.3	1.200	<0.001
AA efficiency							
bc^−1^_Lys_ (×10^6^)	60 ^a^ ± 11	68 ^a^ ± 6	68 ^a^ ± 19	64 ^a^ ± 8	96 ^b^ ± 6	2.667	0.004
bc^−1^_Met_ (×10^6^)	221 ^a^ ± 40	200 ^a^ ± 16	285 ^ab^ ± 78	235 ^a^ ± 28	353 ^b^ ± 59	11.728	<0.001
bc^−1^_Thr_ (×10^6^)	92 ^a^ ± 17	90 ^a^ ± 7	127 ^ab^ ± 35	105 ^a^ ± 12	158 ^b^ ± 10	5.328	<0.001
bc^−1^_Leu_ (×10^6^)	49 ^ab^ ± 9	47 ^a^ ± 4	69 ^bc^ ± 19	55 ^ab^ ± 7	83 ^c^ ± 5	2.900	<0.001
bc^−1^_His_ (×10^6^)	126 ^a^ ± 23	189 ^b^ ± 16	177 ^ab^ ± 49	222 ^bc^ ± 26	245 ^c^ ± 16	8.564	<0.001

BW: body weight; DM: dry matter; NPU_std_: standardized net protein utilization; HM (A): Hermetia meal with Lys (80%); SM (A): Spirulina meal with Lys (80%); HM (AA): Hermetia meal with extended AA supply; SM (AA): Spirulina meal with extended AA supply; SEM: standard error of the mean; *p*: *p*-value; ^a–c^ values within a row with different superscripts are significantly different (*p* < 0.05). ^1^ Outlier (α = 0.2) according to Dixon and Massey [42]. ^2^ Applied for NPU standardization based on: NMR = 388 mg/BW_kg_^0.67^/d; NR_max_T = 3104 mg/BW_kg_^0.67^/d. ^3^ Standardized daily N intake = 3800 mg/BW_kg_^0.67^.
